# Buyang Huanwu Decoction Exerts Cardioprotective Effects through Targeting Angiogenesis via Caveolin-1/VEGF Signaling Pathway in Mice with Acute Myocardial Infarction

**DOI:** 10.1155/2019/4275984

**Published:** 2019-04-16

**Authors:** Jia-Zhen Zhu, Xiao-Yi Bao, Qun Zheng, Qiang Tong, Peng-Chong Zhu, Zhuang Zhuang, Yan Wang

**Affiliations:** Department of Cardiology, The Second Affiliated Hospital and Yuying Children's Hospital of Wenzhou Medical University, Wenzhou 325027, China

## Abstract

**Background:**

Acute myocardial infarction (AMI) remains a leading cause of morbidity and mortality worldwide. The idea of therapeutic angiogenesis in ischemic myocardium is a promising strategy for MI patients. Buyang Huanwu decoction (BHD), a famous Chinese herbal prescription, exerted antioxidant, antiapoptotic, and anti-inflammatory effects, which contribute to cardio-/cerebral protection. Here, we aim to investigate the effects of BHD on angiogenesis through the caveolin-1 (Cav-1)/vascular endothelial growth factor (VEGF) pathway in MI model of mice.

**Materials and Methods:**

C57BL/6 mice were randomly divided into 3 groups by the table of random number: (1) sham-operated group (sham, *n* = 15), (2) AMI group (AMI+sham, *n* = 20), and (3) BHD-treated group (AMI+BHD, *n* = 20). 2,3,5-Triphenyltetrazolium chloride solution stain was used to determine myocardial infarct size. Myocardial histopathology was tested using Masson staining and hematoxylin-eosin staining. CD31 immunofluorescence staining was used to analyze the angiogenesis in the infarction border zone. Western blot analysis, immunofluorescence staining, and/or real-time quantitative reverse transcription polymerase chain reaction was applied to test the expression of Cav-1, VEGF, vascular endothelial growth factor receptor 2 (VEGFR2), and/or phosphorylated extracellular signal-regulated kinase (p-ERK). All statistical analyses were performed using the SPSS 20.0 software and GraphPad Prism 6.05. Values of *P* < 0.05 were considered as statistically significant.

**Results and Conclusion:**

Compared with the AMI group, the BHD-treated group showed a significant improvement in the heart weight/body weight ratio, echocardiography images, cardiac function, infarct size, Mason staining of the collagen deposition area, and density of microvessel in the infarction border zone (*P* < 0.05). Compared with the AMI group, BHD promoted the expression of Cav-1, VEGF, VEGFR2, and p-ERK in the infarction border zone after AMI. BHD could exert cardioprotective effects on the mouse model with AMI through targeting angiogenesis via Cav-1/VEGF signaling pathway.

## 1. Introduction

Based on the fourth edition of universal definition of myocardial infarction (MI), acute MI (AMI) was defined as having clinical evidences of acute myocardial injury with at least one of the following items: clinical symptoms of myocardial ischemia, new ischemic changes in electrocardiogram (ECG), development of pathological Q waves, imaging evidences in accordance with an ischemic aetiology as new loss of surviving myocardium or new regional ventricular wall motion abnormality, and identification of a coronary thrombus by coronary angiography or autopsy [1]. Epidemiological findings from National Health and Nutrition Examination Survey 2011 to 2014 (National Heart, Lung, and Blood Institute tabulation) manifested that the overall prevalence of MI was 3.0% in US adults greater than or equal to 20 years old [2]. Reperfusion and revascularization therapy, including thrombolysis and/or percutaneous coronary intervention (PCI), should be administrated as quickly and effectively as possible to limit infarct size or prevent complete occlusion [3]. Reduction in the mortality rate of AMI is one big success story of modern medicine [3]. However, the process of restoring coronary blood flow to the ischemic myocardium may lead to myocardial ischemia/reperfusion (I/R) injury such as myocardial stunning, no-reflow phenomenon, and reperfusion arrhythmias. Several strategies such as pharmacological treatment and mechanical therapies could reduce I/R injury in animal studies or small-scale clinical trials, but the results are inconclusive [4]. Thus, there is still a need to develop a novel cardioprotective strategy for AMI patients.

Angiogenesis is defined as the growth and proliferation of new blood vessels from preexisting vascular structures [5]. Therapeutic angiogenesis refers to utilizing angiogenic growth factors to increase the growth of collateral blood vessels and promote new vascularization, so as to improve blood flow and tissue perfusion [6]. Promoting angiogenesis in ischemic myocardium that lack sufficient perfusion remains a promising strategy for MI patients [7, 8]. Although there will be a spontaneous angiogenic response in AMI which could partly reestablish blood flow in myocardium, this protective response is usually insufficient to restore the physiological level of tissue perfusion [7]. However, limited medical therapies have yet been proved to be able to successfully promote angiogenesis in AMI patients [9].

Growing evidences have shown that Chinese herbal medicines (CHM) could provide therapeutic effect on AMI by targeting angiogenesis [10–12]. Buyang Huanwu decoction (BHD), originally recorded in *Yilin Gaicuo (Correction on Errors in Medical Classics)* written by Wang in 1830, is a famous Chinese herbal prescription, has been used for the treatment of various vascular diseases in China for hundreds of years [13], and now is still being used in China and other countries around the world. BHD is composed of seven kinds of Chinese herbs (Table 1): (a) huang qi (radix astragali, the dried roots of *Astragalus membranaceus* (Fisch.) Bunge), (b) dang gui (radix angelicae sinensis, the dried lateral roots of *Angelica sinensis* (Oliv.) Diels), (c) chi shao (radix paeoniae rubra, the dried roots of *Paeonia lactiflora* Pall), (d) chuan xiong (rhizoma chuanxiong the dried rhizomes of *Ligusticum striatum* DC), (e) hong hua (flos carthami, the dried flowers of *Carthamus tinctorius* L), (f) tao ren (peach kernel, the dried seeds of *Prunus persica* (L.) Batsch), and (g) di long (Lumbricus, the dried bodies of *Pheretima aspergillum* (E. Perrier)), all of which are recorded in http://www.theplantlist.org and *Chinese Pharmacopoeia*. Based on traditional Chinese medicine theory, BHD has the function of invigorating the body, enhancing blood circulation, and activating Qi flow through energy meridians. Growing evidence has suggested the cardio-/cerebral protective functions of BHD in humans and animal models [14–22]. Recent studies on pharmacology and biochemistry also have shown that the protective functions of BHD on cardiocerebrovascular disease at least in part through the following mechanisms: antioxidant [18, 23, 24], antiapoptosis [25, 26], anti-inflammatory [19, 27], and improving hemorheological disorders [19]. An overview of systematic reviews indicated that BHD could treat a wide range of vascular disorders such as acute ischemic stroke and angina pectoris through targeting vascularity [28]. Studies showed that BHD could promote angiogenesis through increasing the expression of VEGF, VEGFR2, Flk-1, bFGF, and angiopoietin-1 (Ang-1) in ischemic stroke models both in vivo and in vitro [14, 29–33]. The regulation of BHD on the vascular endothelial growth factor (VEGF) signaling pathway according to the pathway enrichment analysis deserves to be studied in order to fully apprehend its latent capacity on treatment and its correlation with angiogenesis [34].

Caveolin-1 (Cav-1), the signature protein of endothelial cell caveolae, is involved in many physiological and pathological processes such as antifibrosis [35], inflammation [36], and oxidative stress [37]. Recent studies [38, 39] have demonstrated that Cav-1 is highly expressed in the vasculature in the process of blood vessel growth and could regulate the angiogenic activity of endothelial cells. Loss of Cav-1 would lead to the inhibition of vessel development and vascular remodeling [40]. Furthermore, Cav-1 plays a pivotal role in the signaling pathway of VEGF/VEGFR2-stimulated angiogenesis and is associated with angiogenic biological activities [41]. Resveratrol, a Cav-1 agonist, significantly elevated eNOS and VEGF protein levels in hypercholesterolemic rats with focal myocardial ischemic injury [42]. These evidences suggested that the Cav-1/VEGF pathway might play a critical role in angiogenesis after myocardial ischemic injury. Thus, in the present study, we aim to investigate the effects of BHD on angiogenesis through the Cav-1/VEGF pathway on the MI model of mice.

## 2. Materials and Methods

### 2.1. Animals

Thirty adult C57BL/6 male mice at 6-8 weeks of age and 20-25 g weight were obtained from Shanghai Slack Laboratory Animal Research Center and housed in the laboratory animal center of Wenzhou Medical University. All the mice were kept under 12 h light/dark cycles, temperature 22 ± 1°C, and provided with food and water ad libitum. The animals used were treated in accordance with the *Guide for the Care and Use of Laboratory Animals*, published by the National Institutes of Health (NIH). The study instructions were approved by the Animal Ethics Committee of the laboratory animal center of Wenzhou Medical University (number wydw2014-0058). All efforts were made to minimize the suffering of animals used.

### 2.2. Drugs and Reagents

BHD which consists of huang qi (radix astragali seuhedysari), dang gui (radix angelica sinensis), chi shao (radix paeoniae rubra), chuan xiong (rhizoma ligustici chuanxiong), hong hua (flos carthami), tao ren (semen persicae), and di long (Lumbricus) with a dispensing ratio of 120 : 6 : 4.5 : 3 : 3 : 3 : 3 was purchased from Sanjiu Medical & Pharmaceutical Co. Ltd., Shenzhen, China (granule preparations, approval number: country medicine accurate character Z44020711); CD31 antibody (ab28364), Cav-1 polyclonal antibody (ab2910), and VEGF polyclonal antibody (ab46154) were purchased from Abcam (UK); extracellular regulated protein kinases (ERK1/2) monoclonal antibody (4695), phosphorylated extracellular regulated protein kinases (p-ERK1/2) monoclonal antibody (4370), glyceraldehyde phosphate dehydrogenase (GAPDH) monoclonal antibody (5174), and vascular endothelial growth factor receptor 2 (VEGFR2) monoclonal antibody (2479) were purchased from Cell Signaling Technology (USA).

### 2.3. AMI Model Establishment

Establishment of the AMI model referred to the previous publication [43]. Briefly, mice were anesthetized by isoflurane, and respiration was assisted with a ventilator (Inspira, Harvard Apparatus, Holliston, MA) in a volume-controlled mode at 80 strokes per minute. After fixation, thoracotomy was done at the 3^rd^ intercostal space via the left lateral chest wall to expose the pericardium and heart. The AMI model was established by perpetually ligating the left anterior descending coronary artery (LAD) in 2 mm from its origin (near the main pulmonary artery) with a 7-0 silk suture, resulting in the development of pale color in the distal part of ligation.

All C57BL/6 mice (*n* = 55) were randomly divided into 3 groups by the table of random number: (1) sham-operated group (sham, *n* = 15), the LAD was encircled by a 7-0 silk suture without ligation; (2) AMI group (AMI+sham, *n* = 20), gavage with 0.2 ml 0.9% normal saline (once a day) 3 days before modeling until 14 days after modeling; and (3) BHD-treated group (AMI+BHD, *n* = 20), gavage with 0.2 ml BHD (20 g/kg, once a day) 3 days before modeling until 14 days after modeling.

### 2.4. Doppler Echocardiography Study

Fourteen days after modeling, mice undergo transthoracic echocardiography by the M-mode transducer (Acuson Sequoia 512, Sonos, Germany) after induction of anesthesia. At the papillary muscle level, M-mode tracings through short-axis view were recorded through the anterior and posterior left ventricle (LV) walls to measure LV end-diastolic dimension (LVEDd), LV end-systolic dimension (LVESd), LV fraction shortening (LVFS), and LV ejective fraction (LVEF) with the Simpson approach. All measurements were done by an experienced doctor who was blinded to the experimental design.

### 2.5. Determination of Myocardial Infarct Size

Euthanasia was done on the mice at 14 days after modeling through intraperitoneal injection of excessive pentobarbital sodium. The heart of the mice was separated from the aortic arch, major blood vessels, and extracardiac connective tissue and rinsed in phosphate-buffered saline to wash away the bloodstain. The heart tissues were semifreezed in a −20°C freezer for 30 minutes and then cut into 5 slices (1 mm thick) in a perpendicular way to the long axis. The slices were incubated in 1% 2,3,5-triphenyltetrazolium chloride solution (TTC) at 37°C for 15 minutes. After carefully evaluating the whole surface area, segments with brick red staining were identified as viable (noninfarcted area) and those without staining were identified as nonviable (infarcted area). Finally, the 3rd slice of each heart was chosen to calculate the infarct size (infarcted area/(noninfarcted area + infarcted area)) by the Image-Pro Plus 6.0 software (Media Cybernetics, Silver Spring, USA).

### 2.6. Myocardial Histopathology

The left ventricle including the region of MI was embedded in paraffin. The samples were then sectioned into 5 *μ*m thick slices. Masson staining and hematoxylin-eosin (HE) staining were applied separately. Morphological changes of nuclei and cytoplasm around the marginal zone of MI in HE staining were observed by an optical microscope (Olympus, Japan). Image-Pro Plus 6.0 software (Media Cybernetics, Silver Spring, USA) was used to calculate the percentage of collagen deposition around the marginal zone of MI to assess the degree of fibrosis in the infarcted myocardium.

### 2.7. Western Blot Analysis

Total protein isolated from the myocardium was separated by SDS-PAGE and transferred to a polyvinylidene difluoride (PVDF) membrane. The membranes were then blocked with 5% fat-free milk and incubated overnight at 4°C with primary antibodies including Cav-1 (1 : 1000), VEGF (1 : 1000), VEGFR2 (1 : 1000), GADPH (1 : 10000), ERK1/2 (1 : 1000), and p-ERK1/2 (1 : 2000). After washing with TBST for three times, the membranes were incubated with secondary antibodies (1 : 10000) for 2 h at room temperature. ChemiDoc™ XRS+ Imaging System was used to visualize the signals. Javas freely available NIH ImageJ software (NIH, Bethesda, MD, USA) was used to quantify the intensity of immune reactivity.

### 2.8. Immunofluorescence Staining

After routine dewaxing and hydration, the antigens in myocardial tissue sections were repaired by sodium citrate buffer at 100°C. After washing thrice with PBS, tissues were treated with 3% hydrogen peroxide for 30 min. 1% bovine serum albumin (BSA) was used to block the antigen. The tissues were then incubated with CD31 antibody (1 : 300), Cav-1 antibody (1 : 500), VEGF antibody (1 : 400), VEGFR2 antibody (1 : 200), and p-ERK antibody (1 : 200) at 4°C and then followed by 60 min of incubation with Alexa Flour 647- or 488-conjugated antibody (1 : 400) at 37°C. To visualize the nuclei, the cells were counterstained with 4′,6-diamidino-2-phenylindole (DAPI) for 5 min in the dark. The images were captured using a fluorescence microscope and then analyzed with the Image-Pro Plus 6.0 software (Media Cybernetics, Silver Spring, USA).

### 2.9. Real-Time Quantitative Reverse Transcription Polymerase Chain Reaction (RT-qPCR)

Total RNA was isolated using the TRIzol reagent (Invitrogen, USA). RNA samples from each group were reverse transcribed into cDNA using the PrimeScript™ RT reagent Kit (TAKARA, Japan). Quantitative RT-qPCR was performed on a LightCycler thermal cycler system (Bio-Rad, USA) using SYBR® Premix Ex Taq™ II (TAKARA, Japan) and gene-specific primers. Gene-specific primers were as follows: Cav-1: forward, 5′-GACCTAATCCAACCATCAT-3′ and reverse, 5′-AGCAAGAACATTACCTCAA-3′; VEGF: forward, 5′-GACTATTCAGCGGACTCA-3′ and reverse, 5′-AAGAACCAACCTCCTCAA-3′; VEGFR2: forward, 5′-AATGATTGTTGGCGATGAA-3′ and reverse, 5′-GTGAGGATGACCGTGTAG-3′; and *β*-actin: forward, 5′-ACCTGCCCTTTAGAACTT-3′ and reverse, 5′-GCTCCAGGGACTATCTTT-3′.

### 2.10. Statistical Analysis

All data were expressed as mean ± standard deviation (SD). Difference between two groups was analyzed using two-tailed Student's *t*-test. Multiple groups were compared using one-way analysis of variance (ANOVA) and followed by LSD post hoc comparisons when appropriate. All statistical analyses were performed using the SPSS 20.0 software and GraphPad Prism 6.05. Values of *P* < 0.05 were considered as statistically significant.

## 3. Result

### 3.1. Effect of BHD on the Survival Rate and the Heart Weight/Body Weight Ratio after AMI

**Figure 1:**
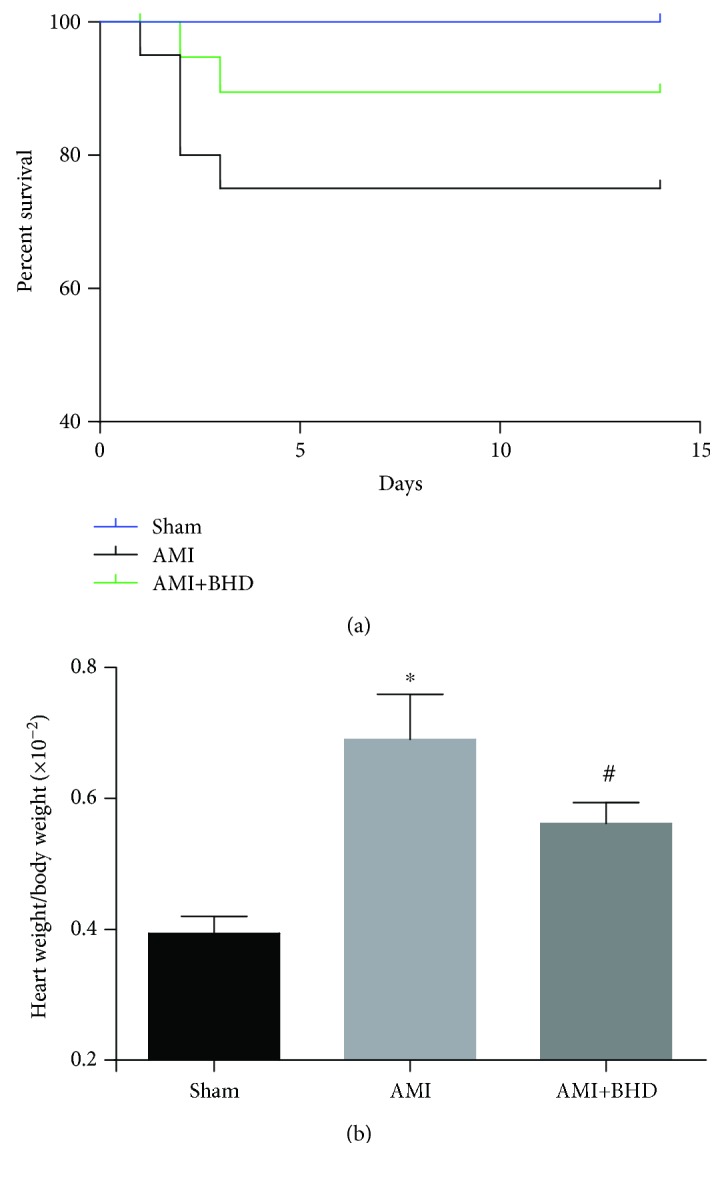
Survival rate and heart weight/body weight ratio at 14 days after AMI in mice (sham: *n* = 15, AMI: *n* = 15, and BHD+AMI: *n* = 18). (a) The survival rate of mice in the BHD-treated group compared with the AMI group (log-rank: *P* = 0.0829); (b) the heart weight/body weight ratio of mice (mean ± SD). ^∗^*P* < 0.05, compared with the sham group; ^#^*P* < 0.05, compared with the AMI group.

After 14 days, all mice in the sham group survived, while the BHD-treated group exhibited a trend towards an improved overall survival rate after the induction of AMI, but differences did not reach statistical significance (log-rank: *P* = 0.0829, Figure 1(a)). The heart weight/body weight ratio was significantly decreased in the BHD-treated group compared with the AMI group (*P* < 0.05, Figure 1(b)).

### 3.2. Effect of BHD on Cardiac Function and Infarct Size after AMI

**Figure 2:**
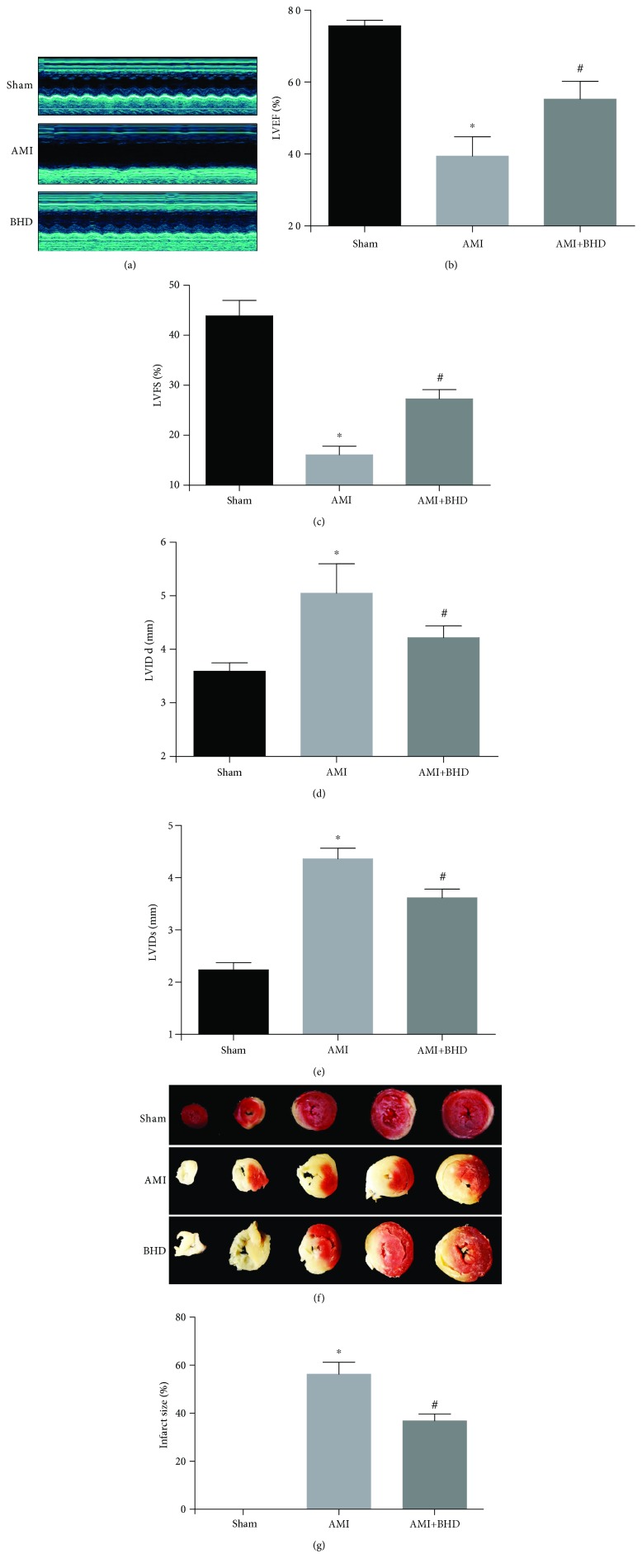
Cardiac function and infarct size at 14 days after AMI in mice. (a) M-mode echocardiographic images of the mice in each group. (b) The analysis of LVEF (*n* = 6). (c) The analysis of LVFS (*n* = 6). (d) The analysis of LVIDd (*n* = 6). (e) The analysis of LVIDs (*n* = 6). (f) Representative image of infarct size by cardiac 2,3,5-triphenyltetrazolium chloride (TTC) staining. (g) The analysis of the infarcted size (sham: *n* = 5, AMI: *n* = 5, and BHD+AMI: *n* = 6). ^∗^*P* < 0.05, compared with the sham group; ^#^*P* < 0.05, compared with the AMI group.

As shown by echocardiography images (Figure 2(a)), there was significant improvement of LVEF in the BHD-treated group (75.65 ± 0.64%) compared with the AMI group (39.40 ± 2.21%) at 14 days after AMI (Figure 2(b)). Significant improvements in cardiac function were also observed in LVFS, LVIDd, and LVIDs (*P* < 0.05). The infarct size in the AMI group was 56.20 ± 2.26% (Figures 2(g) and 2(h)). Compared with the AMI group, the infarct size (36.74 ± 1.22%) was markedly reduced in the BHD-treated group (*P* < 0.05).

### 3.3. Effect of BHD on Histological Changes and Fibrosis in Myocardial Tissue after AMI

**Figure 3:**
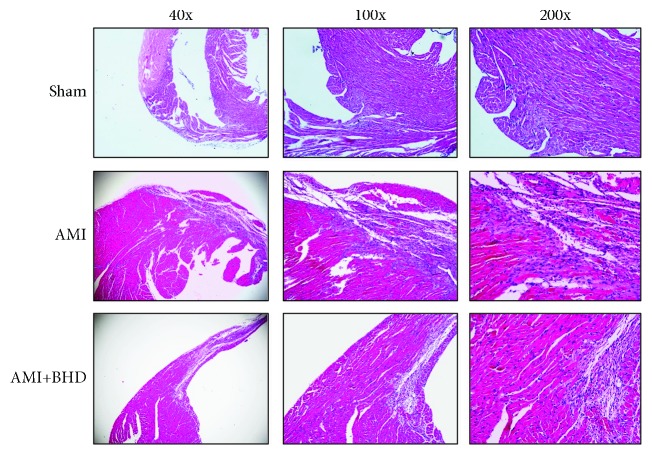
Histological changes in myocardial tissue at 14 days after AMI in mice (sham: *n* = 4, AMI: *n* = 4, and BHD+AMI: *n* = 6).

**Figure 4:**
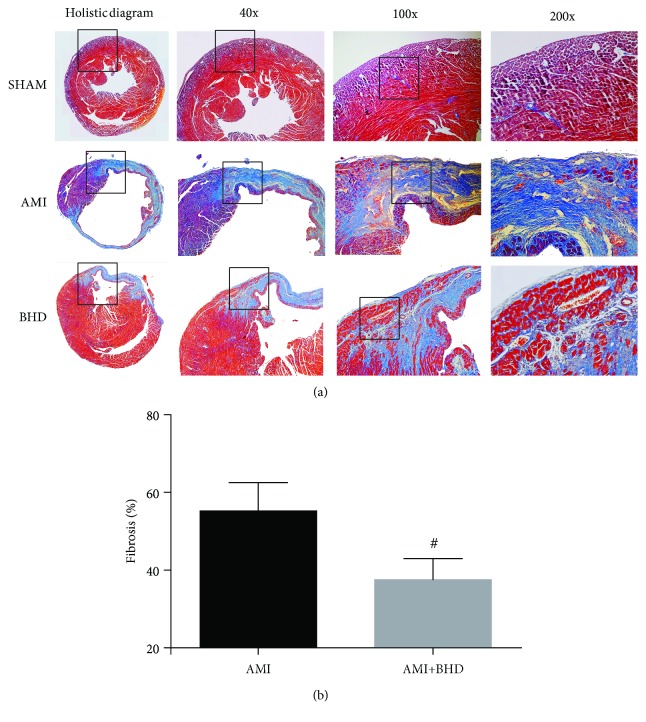
Fibrosis in myocardial tissue at 14 days after AMI in mice (mean ± SD; sham: *n* = 4, AMI: *n* = 4, and BHD+AMI: *n* = 6). (a) Representative images of Masson's trichrome staining. (b) Quantitative analysis of the collagen deposition area. ^#^*P* < 0.05, compared with the AMI group.

By HE staining, the AMI group showed marked necrotic changes in myofibrils with severe infiltration of inflammation and interstitial edema (Figure 3). BHD-treated group exhibited only focal tissue necrosis, mild inflammatory infiltration, and interstitial edema (Figure 3). Compared with the AMI group, Mason staining of the collagen deposition area on myocardial fibrosis was significantly decreased in the BHD-treated group (*P* < 0.05, Figures 4(a) and 4(b)).

### 3.4. Effect of BHD on Angiogenesis after AMI

**Figure 5:**
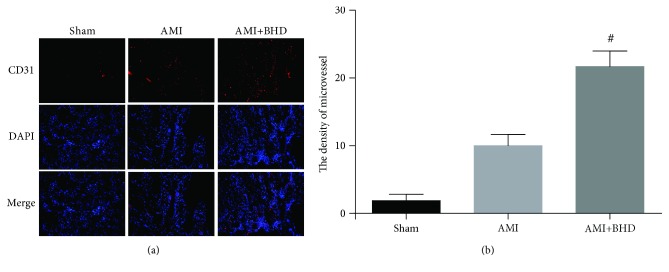
Density of microvessel in the infarction border zone at 14 days after AMI in mice (mean ± SD; sham: *n* = 4, AMI: *n* = 4, and BHD+AMI: *n* = 6). (a) Representative images of CD31 staining. (b) Quantitative analysis of the density of microvessel. ^#^*P* < 0.05, compared with the AMI group.

To verify whether myocardial protection of BHD is associated with angiogenesis in the infarction border zone, the immunohistochemical analysis was performed by CD31 staining. As shown in Figure 5(b), the density of microvessel in the BHD-treated group was much higher than that in the AMI group (*P* < 0.05).

### 3.5. Effect of BHD on Expression of Cav-1, VEGF, VEGFR2, and p-ERK in the Infarction Border Zone after AMI

**Figure 6:**
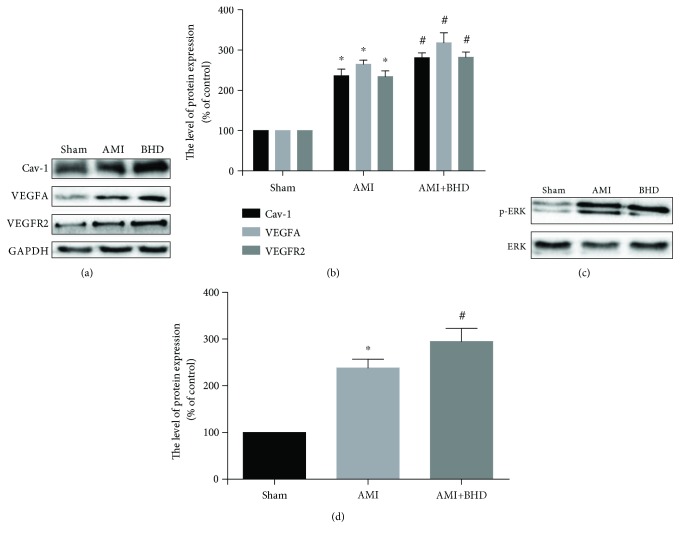
Western blot analysis of Cav-1, VEGF, VEGFR2, and p-ERK1/2 expression in the infarction border zone at 14 days after AMI in mice (mean ± SD, *n* = 6). (a) Western blot analysis of the expression of Cav-1, VEGF, and VEGFR2. (b) Quantitative analysis for the western blot results of Cav-1, VEGF, and VEGFR2. (c) Western blot analysis of the expression of p-ERK1/2. (d) Quantitative analysis for the western blot results of p-ERK1/2. ^∗^*P* < 0.05, compared with the sham group; ^#^*P* < 0.05, compared with the AMI group.

**Figure 7:**
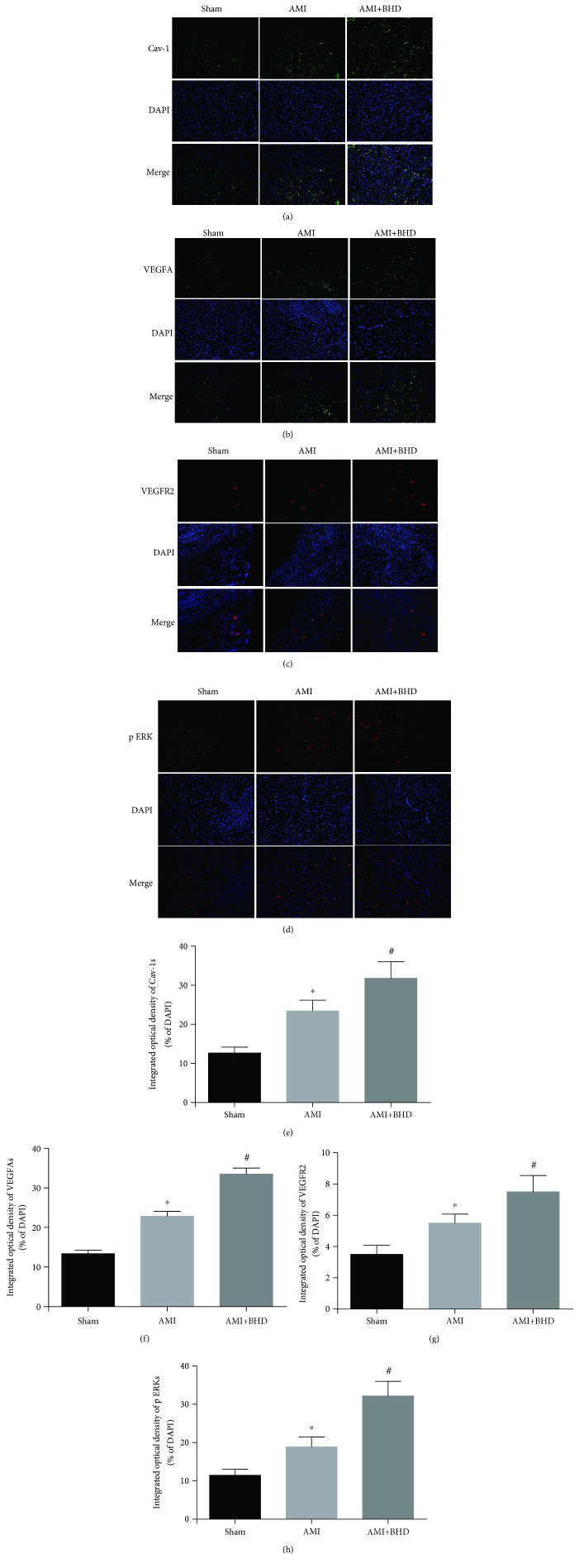
Immunofluorescence staining of Cav-1, VEGF, VEGFR2, and p-ERK1/2 in the infarction border zone at 14 days after AMI in mice (mean ± SD; sham: *n* = 4, AMI: *n* = 4, and BHD+AMI: *n* = 6). (a) Immunofluorescence staining of Cav-1. (b) Immunofluorescence staining of VEGF. (c) Immunofluorescence staining of VEGFR2. (d) Immunofluorescence staining of p-ERK1/2. (e) Quantitative analysis of Cav-1. (f) Quantitative analysis of VEGF. (g) Quantitative analysis of VEGFR2. (h) Quantitative analysis of p-ERK1/2. ^∗^*P* < 0.05, compared with the sham group; ^#^*P* < 0.05, compared with the AMI group.

**Figure 8:**
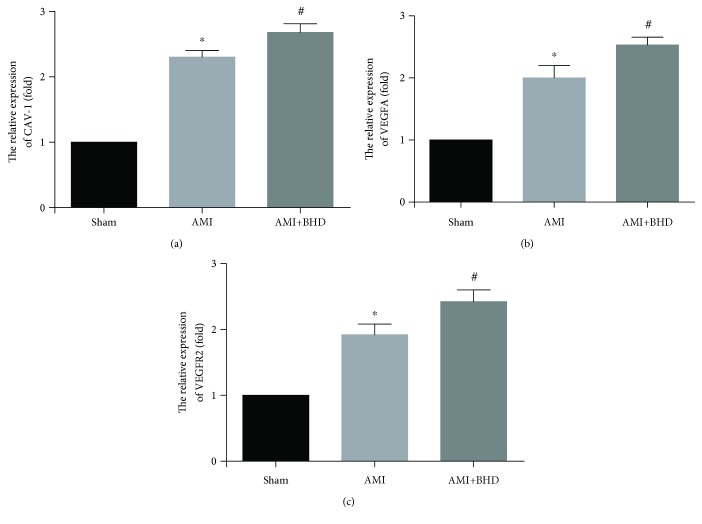
The mRNA expression of Cav-1, VEGF, and VEGFR2 at 14 days after AMI in mice (mean ± SD, *n* = 6). (a) The mRNA expression of Cav-1. (b) The mRNA expression of VEGF. (c) The mRNA expression of VEGFR2. ^∗^*P* < 0.05, compared with the sham group; ^#^*P* < 0.05, compared with the AMI group.

Expression of Cav-1, VEGF, and VEGFR2 was elevated in the AMI group compared with the sham group (*P* < 0.05). Furthermore, the expression of Cav-1, VEGF, and VEGFR2 was further increased in the BHD-treated group compared with the AMI group (*P* < 0.05, Figures 6(a) and 6(b)). BHD treatment promoted the phosphorylation of ERK compared with the AMI group (*P* < 0.05, Figures 6(c) and 6(d)). Immunofluorescence indicated that the integrated optical density of Cav-1 (Figures 7(a) and 7(e)), VEGF (Figures 7(b) and 7(f)), VEGFR2 (Figures 7(c) and 7(g)), and p-ERK (Figures 7(d) and 7(h)) was significantly increased in the BHD-treated group compared with the AMI group (*P* < 0.05). RT-PCR showed that the mRNA level of Cav-1 (Figure 8(a)), VEGF (Figure 8(b)), and VEGFR2 (Figure 8(c)) in the BHD-treated group was significantly increased compared with the AMI group (*P* < 0.05).

## 4. Discussion

During AMI, the damage inflicted on the myocardium results in two processes: ischemia and the following reperfusion (I/R) [44]. The edema/sarcolemma rupture, calcium overload/hypercontracture, mitochondrial dysfunction, proteolysis (caspase, calpain), and apoptosis lead to a large amount of reduction of cardiomyocytes. And, the embolism, vasomotor disorder, leukocyte adherence/infiltration, stasis, and capillary rupture/hemorrhage appeared in coronary vascular caused severe myocardial injury [45]. Thus, cardiovascular protection drugs generally work through one or combined aspects of the above targets. In the present study, BHD reduced the myocardial fibrosis and inflammation, promoted angiogenesis in the infarction border zone via Cav-1/VEGF signaling pathway, then reduced the MI size, and improved the cardiac function. It may be because of these improvements that we finally observed a trend towards an improved overall survival rate of the BHD-treated group.

Cav-1 is a major component of the caveola membrane that is expressed in the majority of differentiated cells [46] and plays an important role in regulating the cellular signal transduction, endocytosis, transcytosis, and molecular transport [47]. The cardioprotective effects of Cav-1 in ischemic heart disease have been well reported [48] in both mouse and human specimens; an increase of Cav-1 in an infarcted area was detected in the early stage of MI [38]. Several studies have shown that the activation or preservation of Cav-1 played a protective role in myocardial I/R injury [49–51]. Subsequently, compared with the wild-type mice, Cav-1^−/−^ mice showed a more severe cardiac dysfunction and a lower survival rate after MI [52]. In Cav-1^−/−^ mice, a low-intensity pulsed ultrasound, which is a potential cardiac protection strategy, presented absent cardioprotective effects after myocardial ischemic injury [38]. Cav-1 is also a vital regulator of vascular endothelial homeostasis which controls angiogenesis and vessel function [53]. The adverse influence on angiogenesis after Cav-1 knockout has been confirmed in multiple disease models, including hindlimb ischemia [54], scleroderma fibroblasts [55], colitis [39], AMI [38], and cerebral ischemia [56]. In the present study, BHD increased angiogenesis and the expression of Cav-1 in the infarction border zone, suggesting that the cardioprotective effect of BHD targeted angiogenesis by Cav-1.

Previous studies also indicated that Cav-1 could reduce infarct volume and promote angiogenesis through the VEGF signaling pathway [57, 58]. Recent studies showed that the expression of Cav-1 and VEGF was significantly decreased after the use of the caveolin-1 inhibitor, resulted in increase in neurological deficit and infarction volume [59–61]. Other studies also confirmed this phenomenon at the genetic level. The ablation of Cav-1 gene in mice could result in an impairment in angiogenesis and reduction of VEGF expression [56, 62]. VEGF is a pivotal regulator of blood vessel formation during embryogenesis and angiogenesis [63]. Lots of evidences have shown that VEGF, through combining with its receptor VEGFR2, could trigger multiple downstream signals such as p-ERK, thereby promoting angiogenesis [64–66]. Taken together, these results indicate that Cav-1 could promote angiogenesis by upregulating the VEGF signaling pathway. The present study indicated that BHD increased the CAV-1, VEGF, VEGFR2, and p-ERK in the infarction border zone, suggesting that BHD could promote angiogenesis through the Cav-1/VEGF pathway.

Herbal formulae, with multicomponents and multitargets, may potentially satisfy the demands of complex disease treatment in an integrated manner. Furthermore, investigation on new molecular targets and principles indicated that a single angiogenic substance might be insufficient for inducing therapeutic angiogenesis [67]. Hundreds of constituents have been identified in BHD such as polysaccharides, astragalosides, and isoflavonoids in radix astragali seuhedysari [68], as well as phthalides and phenolic acids in radix angelicae sinensis and rhizoma ligustici chuanxiong, etc. [69, 70]. Network pharmacology can forecast multiple targets and pathways affected by the active components in TCM formulae. Among them, key targets/signaling pathways might be selected and should be experimentally validated.

## 5. Conclusion

The present study demonstrated that BHD could exert cardioprotective effects on the mouse model with AMI through targeting angiogenesis via Cav-1/VEGF signaling pathway.

## Figures and Tables

**Table 1:** Overview of Buyang Huanwu decoction.

Chinese name	Common name	Latin name/family/medicinal parts	Amount (%)
Huang qi	Radix astragali	Astragalus membranaceus (Fisch.) Bunge/Leguminosae/dried roots	120 g (84.2%)
Dang gui	Radix angelicae sinensis	Angelica sinensis (Oliv.) Diels/Apiaceae/dried lateral roots	6 g (4.2%)
Chi shao	Radix paeoniae rubra	Paeonia lactiflora Pall/Paeoniaceae/dried roots	4.5 g (3.2%)
Chuan xiong	Rhizoma chuanxiong	Ligusticum striatum DC/Apiaceae/dried rhizomes	3 g (2.1%)
Hong hua	Flos carthami	Carthamus tinctorius L/Compositae/dried flowers	3 g (2.1%)
Tao ren	Peach kernel	Prunus persica (L.) Batsch/Rosaceae/dried seeds	3 g (2.1%)
Di long	Lumbricus	Pheretima aspergillum (E. Perrier)/dried bodies	3 g (2.1%)

## Data Availability

The data used to support the findings of this study are available from the corresponding author upon request.
